# The PRIDE Study: Evaluation of online methods of data collection

**DOI:** 10.1111/ppe.12618

**Published:** 2019-12-23

**Authors:** Marleen M. H. J. van Gelder, Peter J. F. M. Merkus, Joris van Drongelen, Jessie W. Swarts, Tom H. van de Belt, Nel Roeleveld

**Affiliations:** ^1^ Department for Health Evidence Radboud Institute for Health Sciences Radboud University Medical Center Nijmegen The Netherlands; ^2^ Radboud REshape Innovation Center Radboud University Medical Center Nijmegen The Netherlands; ^3^ Department of Paediatric Pulmonology Radboudumc Amalia Children's Hospital Radboud University Medical Center Nijmegen The Netherlands; ^4^ Department of Obstetrics and Gynaecology Radboud University Medical Center Nijmegen The Netherlands; ^5^ Radboud Institute for Health Sciences Radboud University Medical Center Nijmegen The Netherlands

**Keywords:** birth cohort, eHealth, epidemiologic methods, internet, pregnancy, PRIDE Study

## Abstract

**Background:**

Large birth cohort studies are extremely valuable in assessing associations between early life exposures and long‐term outcomes. Establishing new birth cohorts is challenging due to declining participation rates. Online methods of data collection may increase feasibility, but have not been evaluated thoroughly.

**Objective:**

The primary objective of the ongoing PRegnancy and Infant DEvelopment (PRIDE) Study is to identify exposures during pregnancy and in early life that may affect short‐term or long‐term health of mother and/or child. In this manuscript, we aimed to evaluate methods of recruitment and online data collection applied.

**Population:**

Dutch women aged ≥18 years in early pregnancy.

**Design:**

Prospective cohort study.

**Methods:**

Initially, only prenatal care providers recruited participants, but alternative recruitment methods were added as a result of disappointing participation rates, including collaboration with “Moeders voor Moeders” (organisation that visits women in early pregnancy) and Facebook advertisements. Data on demographic characteristics, obstetric history, maternal health, life style factors, occupational exposures, nutrition, pregnancy complications, and infant outcomes are primarily collected through Web‐based questionnaires at multiple time points during and after pregnancy. Additional data collection components include paternal questionnaires, blood and saliva sampling, and linkage to medical records.

**Preliminary results:**

By September 2019, 9573 women were included in the PRIDE Study, of which 1.3% completed paper‐based questionnaires. Mean age of the women analysed was 30.6 years, 71.1% had a high level of education, 57.2% were primiparae, and mean gestational age at enrolment was 9.9 (range 3, 37) weeks, with slight differences between recruitment methods. Pregnancy outcome was known for 89.8%. Retention rate at 6 months after the estimated date of delivery was estimated at 70%. Multiple validation studies conducted within the PRIDE Study indicated high data quality.

**Conclusion(s):**

Although challenging and time‐consuming, online methods for recruitment and data collection may enable the establishment of new birth cohort studies.

**Synopsis**

1 **Study question**

How did the methods of recruitment and online data collection implemented in the PRIDE Study, an ongoing national prospective cohort study among pregnant women, perform?

2 **What's already known**

Online methods of recruitment and data collection may increase the feasibility of new birth cohort studies, but these have not been evaluated thoroughly.

3 **What this study adds**

Although challenging with regard to recruitment and retention, it is feasible to collect high‐quality data on large numbers of pregnant women and their offspring using online methods of data collection. Evaluation and validation of the new methods used enhance the value of these data for epidemiologic studies.

## 1 INTRODUCTION

Large birth cohort studies have proven to be extremely valuable for assessing associations between exposures early in life and long‐term outcomes.^1^ Examples of such large‐scale studies that enrolled women in pregnancy with follow‐up into childhood include the Amsterdam Born Children and their Development (ABCD) Study,^2^ Avon Longitudinal Study of Parents and Children (ALSPAC),^3^ Born in Bradford,^4^ the Danish National Birth Cohort (DNBC),^5^ Generation R,^6^ and the Norwegian Mother, Father and Child Cohort Study (MoBa).^7^ Central to these cohorts is participant recruitment between 1991 and 2010, but newly established birth cohorts may provide more insight into possible health risks of relatively new exposures and behaviours, such as e‐cigarette use and low‐carb diets. In an attempt to establish such a new birth cohort, we started the PRegnancy and Infant DEvelopment (PRIDE) Study in 2011.

Unfortunately, the cancellations of the US National Children's Study (NCS; 2014) and the UK Life Study (2015), which aimed to include 80 000‐100 000 infants, may have caused other researchers to refrain from establishing large birth cohort studies in the near future.^8^ Parts of the reasons to cancel these two studies were the low recruitment and participation rates, which declined both in birth cohort studies and in general health‐related research over the last decades.^9^ A major reason for this gradual decline, which became steeper in recent years, is the subjective experience of being too busy by potential study participants.^10^ Therefore, efforts should be undertaken to decrease study participant burden, for example by using modern methods of data collection. Implementation of online data collection methods may increase the feasibility of health‐related studies in general and birth cohort studies in particular. For example, completing a Web‐based questionnaire was reported to take only about half the time needed to answer the same questions in a telephone interview.^11^ However, reports of best practices for recruitment and data collection in the field of paediatric and perinatal epidemiology are scarce.

The PRIDE Study was designed with application and validation of Web‐based questionnaires and other online methods of data collection in mind. The design of the PRIDE Study was published in detail previously.^12^ In the current paper, we evaluate the methods of recruitment and online data collection that are being used in this ongoing study. In addition, we give some recommendations on how to optimise recruitment and data collection in such studies.

## 2 METHODS

### 2.1 Overview, structure, and operations

The primary goal of the PRIDE Study, a prospective cohort study with follow‐up into childhood, is to identify factors and circumstances to which women and their (unborn) children are exposed during pregnancy and in early life that may affect short‐term or long‐term health of the mother and/or the child. The PRIDE Study was initiated by reproductive epidemiologists in collaboration with representatives from many clinical disciplines, including midwifery, obstetrics, neonatology, paediatrics, medical psychology, psychiatry, physiology, human nutrition, and clinical pharmacology. The project team based at the Department for Health Evidence, part of the Radboud Institute for Health Sciences, at the Radboud University Medical Center in Nijmegen, The Netherlands, accommodates data collection, develops datasets, provides scientific input, and ensures confidentiality, privacy, and security of the data. Research using PRIDE Study data is conducted by investigators from the project team as well as by national and international collaborators.

### 2.2 Study population

#### 2.2.1 Eligibility criteria

All Dutch pregnant women aged 18 years or older and able to understand the Dutch language are eligible for participation in the PRIDE Study. Although we aimed to include pregnant women before gestational week 17 only, it appeared to be infeasible to always uphold this criterion during enrolment in practice. Therefore, 3.9% of the participants are ≥17 weeks pregnant at enrolment. Gestational carriers and traditional surrogates are excluded from the PRIDE Study.

#### 2.2.2 Recruitment methods

Based on a pilot study among all midwifery practices and hospitals in the Nijmegen region,^12^ we initially planned to recruit pregnant women nationwide through prenatal care providers only. Participating midwifes and gynaecologists invite pregnant women just before or during their first prenatal care visit, which usually takes place between gestational weeks 8 and 12. As a result of disappointing inclusion rates and new opportunities, however, alternative methods for recruitment of study participants were implemented as well. We partnered with “Moeders voor Moeders” (Mothers for Mothers),^13^ an organisation that collects urine from women between gestational weeks 6 and 16 to extract human chorionic gonadotropin for the production of medication used in fertility treatment. Furthermore, we implemented intermittent Facebook and Google AdWords advertisements,^14^ participated in exhibitions at pregnancy fairs, and placed advertisements in magazines targeted at pregnant women.

### 2.3 Data collection

**Figure 1:**
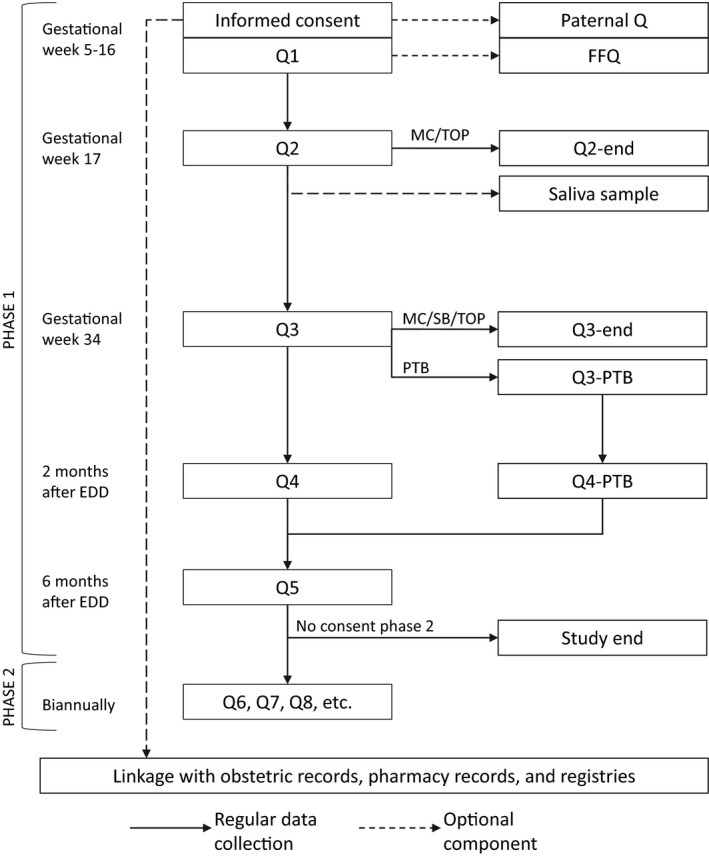
Overview of the standard PRIDE Study data collection. EDD, estimated date of delivery; FFQ, food frequency questionnaire; MC, miscarriage; PTB, preterm birth; Q, questionnaire; SB, stillbirth; TOP, termination of pregnancy

Figure 1 shows an overview of the PRIDE Study data collection. The study consists of two phases: phase 1 entails all data collection components until 6 months after the estimated date of delivery, whereas phase 2 involves biannual questionnaires starting at the age of 1 year until the children reach the age of 21 years. At enrolment, the participating women provide informed consent digitally for use of the self‐reported data with the option to provide consent for linkage to medical records and registries as well.

For subgroups, biological samples are collected as well. Blood samples for genetic and biochemical analyses are collected from participants living in the Nijmegen region only at a mean gestational age of 11.0 weeks (standard deviation [SD] 2.0). In addition, all participants are asked to donate a single saliva sample to measure awakening cortisol levels using an at‐home collection protocol in gestational week 17. Separate paper‐based informed consent is obtained for all biological samples. To facilitate exposure assessment for some specific projects, a number of focus cohorts have been imbedded within the PRIDE Study, including cohorts providing additional information on medication use through diaries and participants who collected multiple faecal samples from themselves and their infants.

#### 2.3.1 Questionnaires

Data for the PRIDE Study are primarily collected using Web‐based questionnaires administered at baseline (gestational weeks 5‐16), in gestational weeks 17 and 34, at 2 and 6 months after the estimated date of delivery, and biannually throughout childhood. The topic lists are provided in Table S1. The questionnaires were constructed based on a number of key exposures and outcomes, using standardised instruments whenever possible (Table 1). For participants who experienced a miscarriage, stillbirth, termination of pregnancy, or preterm birth before completing the follow‐up questionnaire at gestational week 34, adjusted questionnaires are available, tailored to the adverse outcome. Participants receive the invitations to complete each consecutive questionnaire by email, with up to two reminders. Paper‐based questionnaires are available for women who cannot or do not want to participate through the Internet.

**Table 1:** Key exposures and outcomes in the PRIDE Study questionnaires

	Questionnaire 1 (GW 5‐16)	Questionnaire 2 (GW 17)	Questionnaire 3 (GW 34)	Questionnaire 4 (EDD + 2 mo)	Questionnaire 5 (EDD + 6 mo)	Questionnaires 6+ (biannually)
Exposures						
Preconception care	X					
Family history	X					
Maternal anthropometrics	X	X	X	X	X	X
Medication use, including vaccines	X	X	X	X	X	X
Maternal chronic conditions and illnesses	X	X	X	X	X	X
Maternal depression and depressive symptoms	X (HADS, PHQ‐2)	X (EDS, PHQ‐2)	X (HADS, PHQ‐2)	X (EDS, PHQ‐2)	X (EDS, PHQ‐2)	X
Maternal physical and emotional stress	X	X	X	X	X	X
Environmental endocrine disruptors		X				
Occupational exposures		X	X			
Nutrition and vitamin supplements	X	X	X	X	X	
Life style habits	X	X	X	X	X	X
Housing conditions and home environment		X				X
Social determinants	X	X				X
Breast feeding				X	X	
Outcomes						
Pregnancy complications		X	X	X		
Miscarriage		X	X			
Preterm birth			X	X		
Low birthweight/macrosomia			X	X		
Apgar score			X	X		
Developmental delays					X (ASQ)	X (ASQ)
Wheezing, asthma, other respiratory conditions					X	X (ISAAC)
Autism						X (ESAT)
Attention‐deficit/hyperactivity disorder (ADHD)						X
Infectious diseases in childhood					X	X
Obesity (mother and child)				X	X	X
Diabetes (mother and child)					X	X
Hypertension (mother and child)					X	X

Abbreviations: ASQ, Ages and Stages Questionnaire; EDS, Edinburgh Depression Scale; ESAT, Early Screening of Autistic Traits; HADS, Hospital and Anxiety Scale; ISAAC, International Study of Asthma and Allergies in Childhood; PHQ‐2, Patient Health Questionnaire‐2.

In addition to the regular PRIDE Study questionnaires, participating women are given the option to complete a food frequency questionnaire (FFQ) at baseline. The FFQ, which was developed by researchers from Wageningen University, the Netherlands, assesses the dietary intake of a number of macronutrients and micronutrients deemed important in human development. From August 2012 until February 2018, the FFQ was only available as a paper‐based questionnaire. As of March 2018, the FFQ can be completed online.

Furthermore, the women are asked to give permission to send the prospective biological father a single questionnaire focusing on paternal exposures in the 3 months before pregnancy.

#### 2.3.2 Data linkage

Obstetric records are requested to enrich the PRIDE Study database with clinical information, such as blood pressure readings, foetal growth measures, and events during delivery. Furthermore, obstetric records are used to obtain information on pregnancy complications and birth outcomes for participants lost to follow‐up and for questionnaire validation purposes. Likewise, pharmacy records are requested to obtain information on the medications dispensed during pregnancy. We also planned to link the PRIDE Study database to the Perinatal Registry of the Netherlands (Perined) and to other national health registries for specific outcomes in the future.

### 2.4 Statistical analysis

Descriptive statistics were used to characterise the study population. Univariable linear regression models were used to compare continuous maternal characteristics between recruitment methods and modes of data collection (IBM SPSS version 25), whereas Episheet^15^ was used to compare categorical characteristics.

### 2.5 Ethics approval

The PRIDE Study has been approved by the Regional Committee on Research Involving Human Subjects (CMO 2009/305).

## 3 RESULTS

### 3.1 Recruitment

After the pilot phase in the region of Nijmegen, which started in July 2011, recruitment through prenatal care providers was gradually expanded to become nationwide in 2015. Although more than 180 prenatal care providers intended to recruit pregnant women for participation in the PRIDE Study, in reality only approximately 30 midwifery practices enrol participants actively. Combined with the recruitment strategies added in the past few years, a total of 9573 pregnant women were enrolled in the PRIDE Study by 30 September 2019. Due to the diversity of recruitment methods and the accompanying lack of a valid denominator, however, it is impossible to calculate response rates.

Table 2 shows the baseline characteristics of PRIDE Study participants enrolled between July 2011 and December 2018 (N = 8360), stratified by main recruitment method. The mean age of the women included was 30.6 years (SD 3.8), 71.1% had a high level of education, 57.2% were primiparae, and the mean gestational age at enrolment was 9.9 weeks (SD 3.6). Participants recruited through Facebook seemed to be younger (28.8 vs 30.7 years; difference 1.9 years, 95% confidence interval [CI] 1.4, 2.5), to have a lower level of education (43.4% vs 25.4%; difference 17.9%, 95% CI 10.9, 25.0) and higher pre‐pregnancy body mass index (BMI; 26.0 vs 23.6 kg/m^2^; difference 2.4 kg/m^2^, 95% CI 1.9, 3.0), and more likely to be primiparous than participants recruited through prenatal care providers (65.3% vs 54.6%; difference 10.7%, 95% CI 3.9, 17.5). The latter also applied to women recruited through the pregnancy fair (difference 16.0%, 95% CI 9.2, 22.8). The gestational age at enrolment also differed between recruitment methods, varying between 8.2 weeks (SD 2.8) for “Moeders voor Moeders” and 13.4 weeks (SD 3.6) for the pregnancy fair.

**Table 2:** Baseline characteristics of PRIDE Study participants enrolled between July 2011 and December 2018, stratified by the main recruitment methods employed

	All (N = 8360)^a^		Prenatal care providers (July 2011‐2018) (n = 5317)		Moeders voor Moeders (October 2016‐2018) (n = 2383)		Facebook Ads (October 2016‐2018)^b^ (n = 196)		National pregnancy fair (February 2014) (n = 180)	
	n	(%)	n	(%)	n	(%)	n	(%)	n	(%)
Maternal age, years^c^	30.6	(3.8)	30.7	(3.8)	30.8	(3.8)	28.8	(3.7)	29.3	(3.9)
Ethnic background^d^										
Dutch	7218	(86.3)	4604	(86.6)	2052	(86.1)	161	(82.1)	157	(87.2)
Non‐Dutch	733	(8.8)	481	(9.0)	198	(8.3)	13	(6.6)	13	(7.2)
Missing	409	(4.9)	232	(4.4)	133	(5.6)	22	(11.2)	10	(5.6)
Level of education^e^										
Low/intermediate	2047	(24.5)	1352	(25.4)	483	(20.3)	85	(43.4)	61	(33.9)
High	5945	(71.1)	3758	(70.7)	1778	(74.6)	90	(45.9)	111	(61.7)
Missing	368	(4.4)	207	(3.9)	122	(5.1)	21	(10.7)	8	(4.4)
Gravidity										
0 previous pregnancies	3589	(42.9)	2229	(41.9)	1051	(44.1)	85	(43.4)	101	(56.1)
≥1 previous pregnancies	4714	(56.4)	3060	(57.6)	1309	(54.9)	109	(55.6)	77	(42.8)
Missing	57	(0.7)	28	(0.5)	23	(1.0)	2	(1.0)	2	(1.1)
Parity										
0 previous births	4778	(57.2)	2902	(54.6)	1457	(61.1)	128	(65.3)	127	(70.6)
≥1 previous birth	3525	(42.2)	2387	(44.9)	903	(37.9)	66	(33.7)	51	(28.3)
Missing	57	(0.7)	28	(0.5)	23	(1.0)	2	(1.0)	2	(1.1)
Pre‐pregnancy BMI^c^	23.7	(4.2)	23.6	(4.0)	23.9	(4.4)	26.0	(5.3)	23.7	(3.5)
Gestational age at enrolment, weeks^c^	9.9	(3.6)	10.4	(3.4)	8.2	(2.8)	11.2	(5.5)	13.4	(3.6)

Abbreviation: BMI, body mass index.

a Including 284 women not recruited through the main recruitment methods: regular Google search (N = 60), word of mouth (N = 57), unknown (N = 56), previous pregnancy in PRIDE Study (N = 42), other health care professional (N = 24), advertisement in magazine (N = 20), other (N = 19), and Google AdWords (N = 6).

b The advertisement was shown intermittently for a total of 211 d.

c Presented as mean (standard deviation).

d A participant was considered to have a non‐Dutch ethnic background if she or one of her parents was born abroad.^16^

e High level of education: completed higher vocational education or university.

### 3.2 Data availability

**Figure 2:**
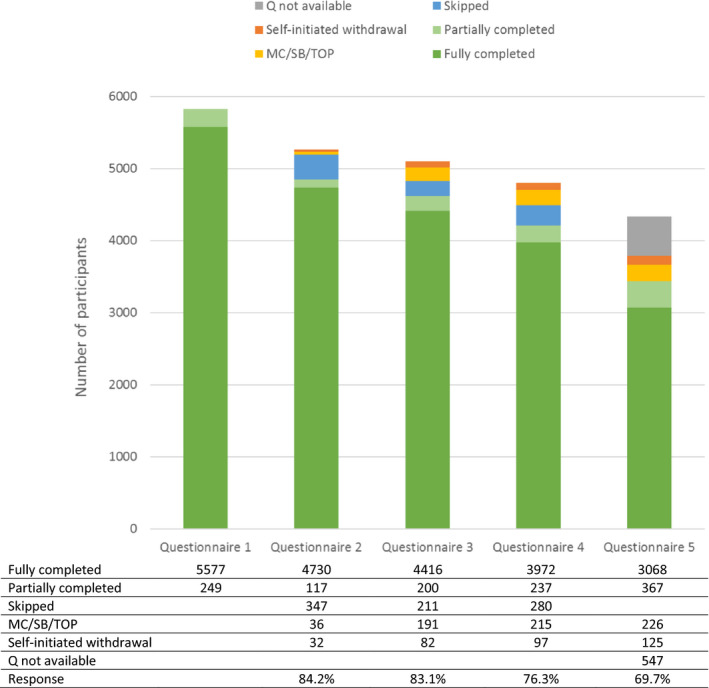
Questionnaire response in phase 1 for PRIDE Study participants with an estimated date of delivery between February 2012 and December 2017. MC, miscarriage; Q, questionnaire; SB, stillbirth; TOP, termination of pregnancy

The currently available dataset for analyses contains all PRIDE Study participants with an estimated date of delivery through 31 December 2017 (n = 5826). Figure 2 shows an overview of the available questionnaires in phase 1 for this population. Follow‐up questionnaire completion rates decreased when the study progressed, ranging between 84.1% for questionnaire 2 (gestational week 17) and 69.7% for questionnaire 5 (6 months after the estimated date of delivery). A total of 5192 participants (89.1%) completed at least one follow‐up questionnaire after baseline. Among those who fully completed questionnaire 5, 96.5% continue the PRIDE Study with the biannual childhood questionnaires in phase 2.

The consent rates for the paternal questionnaire and FFQ were 83.5% and 77.7%, respectively. A total of 2296 prospective fathers (44.5% of those invited) completed the questionnaire, whereas 3570 women completed the FFQ (87.0% of those who consented). The consent rates for data linkage were highest for Perined (84.6%), followed by pharmacy records (75.1%) and obstetric records (70.6%).

Based on the questionnaire data combined with information from obstetric records for those lost to follow‐up, the 5826 pregnancies resulted in 4997 liveborn infants, 200 miscarriages and stillbirths, and 35 terminations of pregnancy. Pregnancy outcome is unknown for 594 pregnancies (10.2%). If we would only rely on self‐reported data, pregnancy outcome would be unknown for 1393 pregnancies (23.9%). The mean gestational age at birth was 39.3 weeks (SD 1.6), the mean birthweight was 3490 g (SD 533), and 51.0% of the liveborn infants were boys.

### 3.3 Web‐based vs paper‐based questionnaires

Due to the high Internet access rates in the Netherlands, only 76 women (1.3%) in the current dataset participated with paper‐based questionnaires. These participants were more likely to have a lower level of education (43.4% vs 25.3%; difference 18.1%, 95% CI 7.0, 29.4) and to have had one or more previous pregnancies (71.1% vs 56.5%; difference 14.6%, 95% CI 4.3, 24.8) or births (67.1% vs 43.9%; difference 23.2%, 95% CI 12.6, 33.9) compared to the women who completed the Web‐based questionnaires (Table 3). Maternal age, ethnic background, and pre‐pregnancy BMI did not seem to differ between the modes of data collection.

**Table 3:** Characteristics of the 5826 PRIDE Study participants in the current analytical dataset, stratified by primary mode of data collection

	Web‐based questionnaires (N = 5750)		Paper‐based questionnaires (N = 76)	
	n	(%)	n	(%)
Maternal age, years^a^	30.5	(3.8)	30.8	(4.0)
Ethnic background^b^				
Dutch	4948	(86.1)	71	(93.4)
Non‐Dutch	529	(9.2)	4	(5.3)
Missing	273	(4.7)	1	(1.3)
Level of education^c^				
Low/intermediate	1453	(25.3)	33	(43.4)
High	4050	(70.4)	42	(55.3)
Missing	247	(4.3)	1	(1.3)
Gravidity				
0 previous pregnancies	2470	(43.0)	22	(28.9)
≥1 previous pregnancies	3250	(56.5)	54	(71.1)
Missing	30	(0.5)	0	(0.0)
Parity				
0 previous births	3196	(55.6)	25	(32.9)
≥1 previous births	2524	(43.9)	51	(67.1)
Missing	30	(0.5)	0	(0.0)
Pre‐pregnancy BMI^a^	23.7	(4.3)	23.8	(3.7)

Abbreviation: BMI, body mass index.

a Presented as mean (standard deviation).

b A participant was considered to have a non‐Dutch ethnic background if she or one of her parents was born abroad.^16^

c High level of education: completed higher vocational education or university.

The larger proportions of item non‐response among women who completed Web‐based questionnaires are largely attributable to partially completed baseline questionnaires, for instance due to quitting halfway through the questionnaire. In that case, the completed sections of the Web‐based questionnaires are saved, whereas partially completed paper‐based questionnaires may be less likely to be returned.

Of the 5414 participants who received a paper‐based FFQ, 4676 (86.4%) returned a completed questionnaire. Between March 2018 and May 2019, 1176 participants received the Web‐based FFQ, of which 948 (80.6%) were returned. Therefore, for reasons yet unknown, the Web‐based FFQ seems slightly less likely to be completed compared with the paper‐based version (relative risk 1.07, 95% CI 1.04, 1.10).

### 3.4 Validation of self‐reported data

In response to the initial lack of evidence on the validity of data obtained by Web‐based questionnaires,^17^ we initiated a series of validation studies within the PRIDE Study on a number of key exposures and outcomes. In general, the validity of data collected through the Web‐based questionnaires was similar or even higher compared to data collected through paper‐based questionnaires and interviews in similar settings. For example, maternal medication use is assessed with a comprehensive indication‐oriented structure with closed‐ended questions to obtain information on generic and brand names, time periods and frequency of use, and quantity taken of prescription and over‐the‐counter medication. This approach was validated with medication diaries as the reference standard, with sensitivity ranging between 0.60 and 0.89 for pregnancy‐related medication groups, between 0.55 and 0.96 for medication groups for chronic conditions, and between 0.30 and 0.70 for medication groups for occasional and short‐term use.^18^ For a number of chronic conditions and allergies, the sensitivity of the Web‐based questionnaire even exceeded the sensitivity of obstetric records, for instance for migraine (0.90 vs 0.40), asthma (0.86 vs 0.61), and hay fever (0.90 vs 0.64), using medical records as reference standard.^19^

In a validation study on birth outcomes, we observed only very small differences between the Web‐based questionnaires and obstetric records for birth outcomes, including gestational age (intraclass correlation coefficient [ICC] 0.91, 95% CI 0.90, 0.92), birthweight (ICC 0.96, 95% CI 0.95, 0.96), birth length (ICC 0.90, 95% CI 0.87, 0.92), and head circumference (ICC 0.88, 95% CI 0.80, 0.93).^20^ Likewise, very few false‐positive and false‐negative reports were observed for gestational diabetes and preeclampsia, but the validity of gestational hypertension, although in range with previous studies, seemed to be lower due to relatively high numbers of false‐positive reports (submitted for publication). Additional validation studies of maternal report of childhood outcomes are being planned.

## 4 COMMENT

### 4.1 Principal findings

Although the number of participants stayed far below expectations,^12^ we showed that it is still feasible to establish a large birth cohort study with over 9500 participants in the current era of declining response rates. Detailed information on key exposures and outcomes was mainly obtained through the use of Web‐based questionnaires, which appear to yield highly accurate data. The first studies based on data from the PRIDE Study have been published.^21^, ^22^, ^23^, ^24^, ^25^, ^26^, ^27^, ^28^

### 4.2 Strengths of the study

One of the major differences between the PRIDE Study and the cancelled NCS and Life Study is the sampling method: we applied a non‐probability sampling approach, whereas the other two studies aimed to be representative for the underlying source population (ie national probability sampling).^29^, ^30^ Indeed, we have an overrepresentation of highly educated women within the PRIDE Study, while women with a non‐Dutch ethnic background seem to be underrepresented. The study population became somewhat more diverse after the implementation of Facebook Ads for recruitment purposes, but only a minority of study participants was recruited through this method. Comparable to previous studies,^31^, ^32^ the study's mixed mode design (ie offering Web‐based and paper‐based questionnaires) also yielded a more diverse population compared with not offering a paper‐based version. Although only few participants requested paper‐based questionnaires, we will keep offering this method of data collection to increase diversity, despite it being labour‐intensive.

Concerns regarding the validity and reliability of data collected through Web‐based questionnaires, which were raised in the beginning of this century,^33^, ^34^ may have led to restraints in the application of this method of data collection in epidemiologic research. Validation studies conducted within the PRIDE Study and in several other settings,^35^, ^36^, ^37^, ^38^, ^39^, ^40^, ^41^ however, indicate that the quality of data obtained with Web‐based questionnaires is certainly sufficient. Partially completed questionnaires may also be considered a proxy for the quality of the questionnaire. Within the PRIDE Study, 4.3% did not finish the baseline questionnaire, which seems to be lower compared with other studies focusing on pregnancy planners (PRESTO: 7.4%)^42^ and pregnant women (NINFEA birth cohort: 8.7%).^43^

### 4.3 Limitations of the data

The non‐probability sampling approach prohibits us from calculating national prevalence estimates for exposures and outcomes. However, the PRIDE Study does not aim to provide these figures, but focuses on providing valid estimates for associations between exposures during pregnancy and early life and maternal and child health outcomes. Reassuringly, previous studies indicated that self‐selection does not bias the exposure‐outcome associations estimated from birth cohort studies.^44^, ^45^, ^46^

In an overview of nine Internet‐based cohorts, Bajardi et al^47^ observed participant follow‐up rates of 43%‐89%, with a median of 63%. For PRESTO, a North American Internet‐based preconception cohort study, a follow‐up rate of 79% in the third trimester was reported.^42^ Therefore, the PRIDE Study's follow‐up rates (76% for pregnancy outcomes and 70% for questionnaire 5 administered 6 months after the estimated date of delivery) are well in line with expectation, especially considering that the participants already enrol in early pregnancy. Although selection bias due to non‐participation in follow‐up questionnaires seems to be limited when factors associated with participation were accounted for in the analyses,^48^ low retention rates are detrimental to statistical power, in particular for long‐term outcomes. Therefore, the ability to obtain information from medical records for outcome assessment for a large proportion of the study population is a major strength of the PRIDE Study design. Although linkage to medical records and registries in cohort studies is valuable for obtaining clinical data that are virtually impossible to collect with self‐reported modes of data collection and for outcome assessment among those lost to follow‐up, linkage cannot replace questionnaires for many other study variables, such as life style factors and occupational exposures.

### 4.4 Interpretation

Summarising the above, our recommendations to others who are considering starting a birth cohort study are to thoroughly consider different participant recruitment strategies. At first, we solely relied on traditional recruitment through health care providers. We observed that some minor protocol adjustments, including more personal contact and provision of a small monetary token of appreciation for each participant to recruiting health care providers while keeping the burden as low as possible, resulted in modest boosts in inclusion rates. Nevertheless, research will never become a priority in clinical settings and competing studies also impact recruitment results. The non‐traditional methods of recruitment not only added more inclusions, but also more diversity to the PRIDE Study population, especially in terms of maternal level of education. In terms of absolute numbers, however, the contribution of the online recruitment methods was limited and needs further refinement and extension, for example through optimal budget settings and addition of other social media.^14^ Furthermore, we are grateful for the collaboration with “Moeders voor Moeders,” but are unaware of similar initiatives in other countries.

As we were among the first to implement Web‐based questionnaires for data collection in health‐related research, we obtained some unique insights into the do's and don'ts concerning this method. Setting up and maintaining a good online system for recruitment and data collection takes a lot of time and effort. Although technical hassles should be prevented, they are unavoidable and directly impact questionnaire completion and study retention rates. We also put major efforts in the look‐and‐feel and user‐friendliness of the questionnaires, taking the strict regulations on privacy and security of data into account. This may have contributed in the relatively low proportion of partially completed questionnaires. In phase 2, we decided to administer multiple shorter questionnaires biannually instead of a more lengthy annual questionnaire based on previous experiences that more regular contact with the study population increases retention. We were pleasantly surprised by the results of the validation studies performed so far, which indicated a very high data quality. Lastly, we learned that a substantial proportion of participants, in particular pregnant women and young mothers, prefer to complete the Web‐based questionnaires on smartphones, which necessitates additional requirements in programming.

## 5 CONCLUSIONS

Enrolment and follow‐up for the PRIDE Study are still ongoing and will be in the upcoming years. This provides us with the opportunity to incorporate other novel methods of data collection, such as mobile applications and wearables, within this cohort to collect even more detailed, timely, and clinically relevant data, which are impossible to obtain through traditional data collection methods. Statistical approaches to deal with the multitude of time‐varying exposures and time‐varying confounders will be applied to assess associations of *in utero* and early life exposures with maternal and child health outcomes. Based on the current inclusion rate, we expect to enrol the 10 000th participant by the beginning of 2020. Ultimately, the insights obtained from the PRIDE Study may be used to improve maternal and child health by developing and implementing preventive measures in preconception and prenatal care as well as during childhood.
