# Supplementary material for: The PRIDE Study: Evaluation of online methods of data collection
Source: Paediatr Perinat Epidemiol. 2019 Dec 23;34(5):484–94. doi: 10.1111/ppe.12618 (PMC7496449; doi:10.1111/ppe.12618)
Supplement: Supplementary file 1 [file PPE-34-484-s001.pdf]

eTable 1. Topics addressed in the PRIDE Study questionnaires.

Phase 1 (prenatal – 6 months postpartum)

Instrument	Timing	General Items	Remarks
Questionnaire 1	Enrolment	• Time to pregnancy / fertility treatment<br>• Natural menstrual cycle*<br>• Pregnancy history<br>• Preconception care<br>• Medical history<br>• Urine incontinence*<br>• Early pregnancy complications<br>• Medication use<br>• Vitamin supplement use<br>• Lifestyle, including caffeine, alcohol, smoking, e-cigarettes, illicit drugs<br>• Major life events<br>• Hospital Depression and Anxiety Scale<br>• PHQ-2<br>• Working conditions (brief)<br>• Physical activity / sleep<br>• Demographics	Focuses on periconceptional period
Paternal questionnaire	Baseline	• Demographics<br>• Medical history<br>• Health and medication use in 3 months before index pregnancy<br>• Vitamin supplement use<br>• Lifestyle, including caffeine, alcohol, smoking, e-cigarettes, illicit drugs<br>• Working conditions<br>• Mobile phone use<br>• Physical activity<br>• Endocrine disruptors	Invited by prospective mother
FFQ	Baseline	Food frequency questionnaire to assess intake of energy, protein, carbohydrates, alcohol, total fat, saturated fat, monounsaturated fat, polyunsaturated fat, linoleic acid, trans fat, cholesterol, α-linolenic acid, eicosapentaenoic acid, docosahexaenoic acid, fibers, vitamins B6, B12, B1, B2, D, C, E, folic acid equivalents, total folate, retinol, RAE, beta-carotene, calcium, lycopene, iron, zinc	Optional
Questionnaire 2	GW 17	• Pregnancy complications<br>• Intentions delivery and breastfeeding<br>• PRAQ-R<br>• Living conditions<br>• Physical activity / sleep<br>• Working conditions, including exposures and work-related stress<br>• Major life events<br>• Edinburgh Depression Scale	Shortened questionnaire for miscarriages / TOPs

		• Illnesses<br>• Medication use<br>• PHQ-2<br>• Vitamin supplement use<br>• Lifestyle, including caffeine, alcohol, smoking, e-cigarettes, illicit drugs<br>• Mobile phone use	
Questionnaire 3	GW 34	• Pregnancy complications<br>• Delivery complications grandmother*<br>• Prenatal diagnostics<br>• Satisfaction with care<br>• Expectations delivery<br>• Breastfeeding intentions (extended)<br>• Working conditions (brief)<br>• Physical activity / sleep<br>• Major life events<br>• Hospital Anxiety and Depression Scale<br>• PHQ-2<br>• Health and illnesses<br>• Medication use<br>• Urine incontinence*<br>• Vitamin supplement use<br>• Lifestyle, including caffeine, alcohol, smoking, e-cigarettes, illicit drugs	Shortened questionnaire for miscarriages / stillbirths / TOPs
Questionnaire 4	EDD + 2 mo	• Delivery<br>• Pregnancy outcome<br>• Infant health<br>• Vaccination<br>• Feeding practices<br>• Infant sleep<br>• Infant crying<br>• Gestational weight gain<br>• Pregnancy complications<br>• Illnesses<br>• Medication use<br>• Major life events<br>• Edinburgh Depression Scale<br>• PHQ-2<br>• Sleep<br>• Vitamin supplement use<br>• Lifestyle, including caffeine, alcohol, smoking, e-cigarettes, illicit drugs	Shortened questionnaire for stillbirths / deceased infants
Questionnaire 5	EDD + 6 mo	• Infant health<br>• Feeding practices<br>• Infant sleep<br>• Infant crying<br>• Ages & Stages Questionnaire<br>• Milestones<br>• Care giving	

		• Major life events<br>• Edinburgh Depression Scale<br>• PHQ-2<br>• Sleeping<br>• Maternal health<br>• Maternal medication use (breastfeeding only)<br>• Urine incontinence*<br>• Maternal vitamin supplement use (breastfeeding only)<br>• Lifestyle, including alcohol, smoking	

EDD, estimated date of delivery; FFQ, food frequency questionnaire; GW, gestational week; TOP, termination of pregnancy
* Collected until March 2018

Phase 2 (1 year – 21 years) – STARTED MARCH 2018

Instrument	Timing	General Items	Remarks
Questionnaire 6	1 year	• Infant health<br>• Rashes<br>• Vaccination<br>• Ages & Stages Questionnaire<br>• Early Screening of Autistic Traits Questionnaire<br>• Milestones<br>• Experiences<br>• Maternal health<br>• Living environment	
Questionnaire 7	1.5 years	• Infant health<br>• Food allergies<br>• Hearing / seeing<br>• Vaccination<br>• Teething<br>• Ages & Stages Questionnaire<br>• Milestones<br>• Infant food<br>• Maternal health	
Questionnaire 8	2 years	• Infant health<br>• ISAAC questionnaire<br>• Ages & Stages Questionnaire<br>• Early Screening of Autistic Traits Questionnaire<br>• Infant habits<br>• Maternal health<br>• Living environment	
Questionnaire 9	2.5 years	• Infant health<br>• Ages & Stages Questionnaire<br>• Milestones<br>• Maternal health	
Questionnaire 10	3 years	• Infant health<br>• ISAAC questionnaire<br>• Ages & Stages Questionnaire	

		• Early Screening of Autistic Traits Questionnaire<br>• Milestones<br>• Maternal health<br>• Living environment	
Questionnaire 11	3.5 years	• Infant health<br>• Hearing<br>• Convulsions<br>• Infant behavior<br>• Milestones<br>• Maternal health	
Questionnaire 12	4 years	• Infant health<br>• ISAAC questionnaire<br>• Diagnosis ADHD/autism<br>• Eye and hair color<br>• Age & Stages Questionnaire<br>• Early screening of Autistic Traits Questionnaire<br>• Maternal health<br>• Living environment	
Questionnaire 13	4.5 years	• Infant health<br>• Accidents<br>• Dental heath<br>• Infant washing<br>• Maternal health	
Questionnaire 14	5 years	• Infant health<br>• ISAAC questionnaire<br>• Ages & Stages Questionnaire<br>• Speech and Language Assessment Scale<br>• Maternal health<br>• Living environment	
Questionnaire 15	5.5 years	• Infant health<br>• Parental locus of control (PLOC)<br>• Satisfaction with Life Scale (SWLS)<br>• General Self-Efficacy Scale (GSE)<br>• IPIP Big-Five factor markers<br>• Maternal health	
Questionnaire 16	6 years	• Infant health – extended<br>• ISAAC questionnaire<br>• Allergies<br>• Maternal health<br>• Adult ADHD Self-Report Scale (ASRS screener)<br>• Living environment	
Questionnaire 17	6.5 years	• Infant health<br>• Eating – Dietary History Questionnaire (mod.)<br>• Maternal health	
Questionnaire 18	7 years	• Infant health<br>• ISAAC<br>• Dental health<br>• Physical activity<br>• Maternal health	

		• Living environment	
Questionnaires 19+		Under construction	
